# Two-dimensional Co-Seismic Surface Displacements Field of the Chi-Chi Earthquake Inferred from SAR Image Matching

**DOI:** 10.3390/s8106484

**Published:** 2008-10-21

**Authors:** Jun Hu, Zhi-Wei Li, Xiao-Li Ding, Jian-Jun Zhu

**Affiliations:** 1 School of Info-Physics and Geomatics Engineering, Central South University, Changsha 410083, Hunan, P.R. China; E-mails: 36185443@163.com; zjj@mail.csu.edu.cn; 2 Department of Land Surveying and Geo-Informatics, The Hong Kong Polytechnic University, Hung Hom, Kowloon, Hong Kong, P.R. China; E-mail: lsxlding@polyu.edu.hk

**Keywords:** Chi-Chi earthquake, Differential Synthetic Aperture Radar (DInSAR), amplitude image match, GPS, Two dimensional (2D) displacements

## Abstract

The *Mw*=7.6 Chi-Chi earthquake in Taiwan occurred in 1999 over the Chelungpu fault and caused a great surface rupture and severe damage. Differential Synthetic Aperture Radar Interferometry (DInSAR) has been applied previously to study the co-seismic ground displacements. There have however been significant limitations in the studies. First, only one-dimensional displacements along the Line-of-Sight (LOS) direction have been measured. The large horizontal displacements along the Chelungpu fault are largely missing from the measurements as the fault is nearly perpendicular to the LOS direction. Second, due to severe signal decorrelation on the hangling wall of the fault, the displacements in that area are un-measurable by differential InSAR method. We estimate the co-seismic displacements in both the azimuth and range directions with the method of SAR amplitude image matching. GPS observations at the 10 GPS stations are used to correct for the orbital ramp in the amplitude matching and to create the two-dimensional (2D) co-seismic surface displacements field using the descending ERS-2 SAR image pair. The results show that the co-seismic displacements range from about -2.0 m to 0.7 m in the azimuth direction (with the positive direction pointing to the flight direction), with the footwall side of the fault moving mainly southwards and the hanging wall side northwards. The displacements in the LOS direction range from about -0.5 m to 1.0 m, with the largest displacement occuring in the northeastern part of the hanging wall (the positive direction points to the satellite from ground). Comparing the results from amplitude matching with those from DInSAR, we can see that while only a very small fraction of the LOS displacement has been recovered by the DInSAR mehtod, the azimuth displacements cannot be well detected with the DInSAR measurements as they are almost perpendicular to the LOS. Therefore, the amplitude matching method is obviously more advantageous than the DInSAR in studying the Chi-Chi earthquake. Another advantage of the method is that the displacement in the hanging wall of the fault that is un-measurable with DInSAR due to severe signal decorrelation can almost completely retrieved in this research. This makes the whole co-seismic displacements field clearly visible and the location of the rupture identifiable. Using displacements measured at 15 independent GPS stations for validation, we found that the RMS values of the differences between the two types of results were 6.9 cm and 5.7 cm respectively in the azimuth and the range directions.

## 1. Introduction

On 21 September 1999, a *Mw*=7.6 earthquake occurred near Chi-Chi Town in Taiwan. The devastating earthquake was triggered by the reactivation of the north-south-trending Chelungpu fault and caused an approximately 80 km long surface rupture along the Chelungpu fault [1]. More than 2,000 people died in the earthquake and 53,551 buildings were destroyed [2].

Differential Interferometic Synthetic Aperture Radar (DInSAR) has been used to measure the co-seismic displacements of the earthquake. Pathier *et al.* [3] found about 10 interferometric fringes in a differential interferogram spanning the earthquake. The fringes are equivalent to about 0.28 m surface displacements in the LOS direction at the footwall of the Chelungpu fault. Liu *et al.* [4, 5] reported that the largest LOS displacements in the footwall of the Chelungpu fault calculated from an averaged interferogrm was about 0.33 m. In these DInSAR studies however, only one-dimensional displacement along the radar Line-of-Sight (LOS) direction were determined. As the Chelungpu fault strike is nearly parallel to the azimuth direction (with an angle of about 12°), the LOS measurements are insensitive to the displacements along the fault strike. In addition, the severe loss of coherence (coherence is defined as the amplitude of the complex correlation coefficient between two patches of co-registratered SAR images, see e.g. [6]) on one side of the fault make it very difficult to determine the displacements with the DInSAR method in the area. Global Positioning System (GPS) has also been used to study the displacements caused by the earthquake although it offers much lower spatial resolution compared with that of the InSAR method. For example, Yu *et al.*[7] reported that 2.4-10.1 m horizontal displacements and 1.2-4.4 m vertical displacements were observed with GPS across the Chelungpu fault. In general, displacements of such magnitudes are difficult to be measured with C- or X-band DInSAR method as the displacements may cause mis-registrations of images and result in fringe rates exceeding the saturation threshold of half a fringe per pixel, which will lead to severe signal decorrelation [8, 11]. Such disadvantages of the DInSAR method have been the motivation to study the ground displacements of the earthquake with the SAR amplitude image matching [8-10] and SPOT image matching methods [22].

We will first present results of co-seismic ground deformation measurements from DInSAR method, followed by those from the method of SAR amplitude images matching. By combining the Azimuth Offset (AZO) and the Range Offset (RO) of the SAR amplitude images, a two-dimensional (2D) surface displacement fields associated with the earthquake will be generated. The results will be compared with GPS observations at some GPS stations in the study area.

## 2. Data Analysis

### 2.1 Analysis of Co-Seismic Interferogram

**Figure 1.**
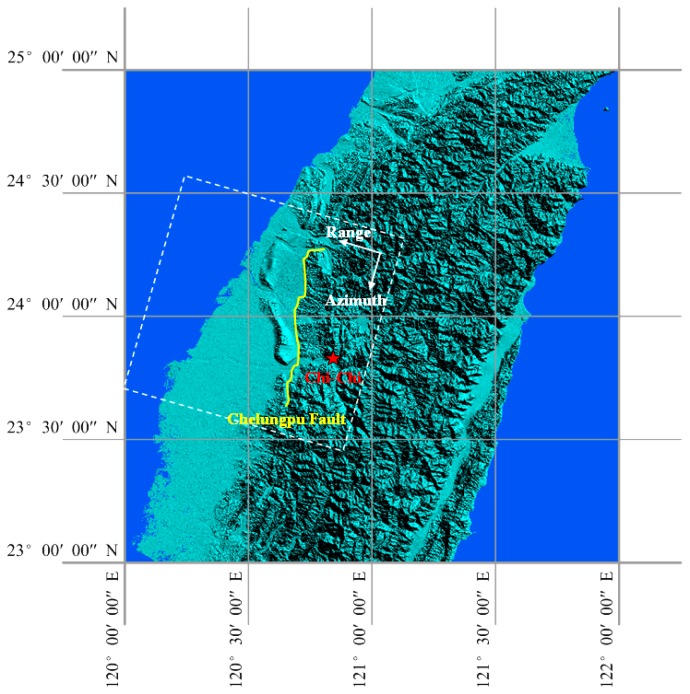
Location of the Chi-Chi earthquake in the WGS 84 system (all figures presented in this paper are in the same reference system except otherwise stated). The red star represents the position of the epicenter. The yellow line represents the Chelungpu fault [7]. The white rectangle indicates the area covered by the ERS-2 descending SAR images used in this study.

Two C band (*λ* = 5.6 cm) ERS-2 satellite SAR images (Table 1) are used to study the co-seismic ground displacements of the Chi-Chi earthquake. The angle of incidence of the radar sensor is about 23 degrees, and the satellite revisit time is 35 days. The images are both from descending orbit, one acquired before the earthquake and the other after the earthquake. Although some ascending SAR images are also available, their time spans are too long to produce meaningful interferograms or offset images. They are therefore not used for this study. The location of the descending SAR images is shown in Figure 1.

**Figure 2.**
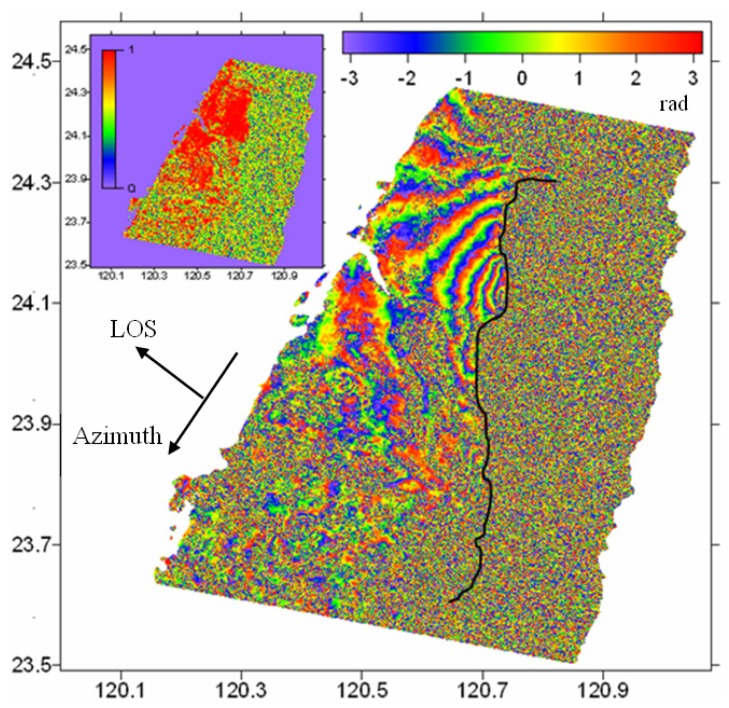
Co-seismic interferogram of Chi-Chi earthquake. Each interferometric fringe represents 2.8 cm of relative displacement in the radar LOS direction. The black line represents the Chelungpu fault. The inset diagram shows the corresponding coherence map. The value zero means that the signals are completely decorrelated and value unity fully coherent.

The SAR images are first processed with the two-pass DInSAR method and the GAMMA software [12]. The perpendicular baseline is about 223 m and the ambiguity height is about 43 m. The 3 arc-second DEM data from the Shuttle Radar Topography Mission (SRTM) is used to remove the topographic phase [13]. Precise ERS-2 orbits from the Delft University of Technology are used in processing the data to reduce errors associated with image co-registration and flat earth phase removal [14-15]. The differential interferogram is then filtered with the improved Goldstein Filter to reduce the phase noises [16]. Finally the co-seismic interferogram of the earthquake is obtained as shown in Figure 2.

It can be seen from Figure 2 that there are about 7-8 fringes in the footwall side of the Chelungpu fault that are equivalent to about 21 cm of total relative radar range changes. Comparing with GPS results reported by Yu *et al.* [7], i.e., 2.4-10.1 m horizontal displacements and 1.2-4.4 m vertical displacements across the Chelungpu fault, it is known that only a very small fraction of the co-seismic displacements have been captured by the DInSAR method. In addition, no good DInSAR results on the hanging wall side of the Chelungpu fault have been reported as the area is covered by forest and serious decorrelaton has been experienced in the area [5, 17].

### 2.2 Offsets Derived from Co-Seismic Amplitude Images

A SAR image contains the phase as well as the amplitude information. We will in this study estimate the co-seismic displacements of the Chi-Chi earthquake with the method of SAR amplitude image matching. The principles of the method are based on the considerations that the post-seismic SAR amplitude image will have pixel-by-pixel shifts with respect to pixels of the pre-seismic SAR amplitude image and that the shift values can be determined through a correlation analysis. The shift values are directly related to the ground displacements caused by the earthquake. The shifts are in general estimated in two orthogonal directions, i.e., the azimuth and range directions, and are correspondingly called AZO and RO, respectively [18]. The amplitude image matching method measures sub-pixels position shifts and can in general achieve an accuracy of about 1/32 pixel [18].

To determine AZO and RO, the amplitude images first need to be co-registered. The co-registration will be implemented in two steps, i.e., coarse and fine co-registration. Preliminary offsets in the azimuth and range directions can be determined in the coarse co-registration. Finer scale offsets can then be calculated in the step of fine co-registration. The AZO and RO between the two amplitude images will be measured in their original sampling space, i.e., about 5 m in azimuth and 8 m in slant range directions. The window size used for estimating the correlation is 64 × 64 pixels. The accuracy of the measurements is about 15 cm in the azimuth direction and 25 cm in the slant range direction. For each pixel, the offset is [19]:

(1) $$R_{\text{offset}}=R_{\text{orbit}}+R_{\text{defo}}$$

where *R_offset_* is related to the AZO and RO between the two images; *R_orbit_* represents the non-coseismic component that is due to the difference in the imaging geometries; *R_defo_* represents the surface displacement in the azimuth or range directions.

**Figure 3.**
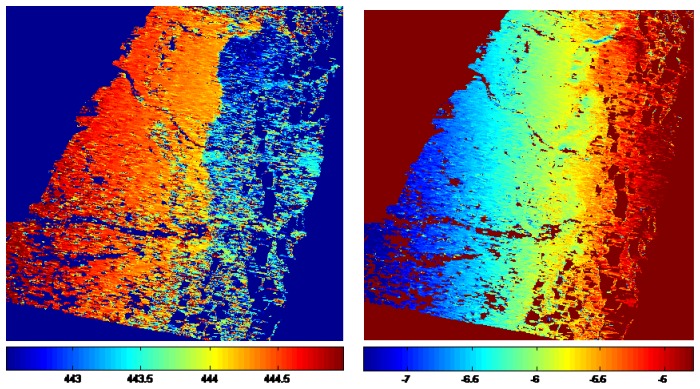
The estimated of AZO (left) and RO (right). Unit: m.

Figure 3 shows the calculated AZO and RO between the SAR images list in Table 1. Although there are significant systematic offsets, the trends of the displacements caused by the earthquake can be clearly seen.

The non-coseismic components due to the difference in the imaging geometries in Equation (1) needs to be removed in order to get the surface displacements. Wang *et al.* [20] suggested to model the non-coseismic components using a bilinear equation:

(2) $$R_{\text{orbit}}=a_{0}+a_{1}x+a_{2}y+a_{3}\text{xy}$$

where *x* and *y* are the coordinates of the pixels in WGS 84 coordinate system, and *a*_0_ , *a*_1_ , *a*_2_ and *a*_3_ are coefficients accounting for the difference in the imaging geometries.

**Figure 4.**
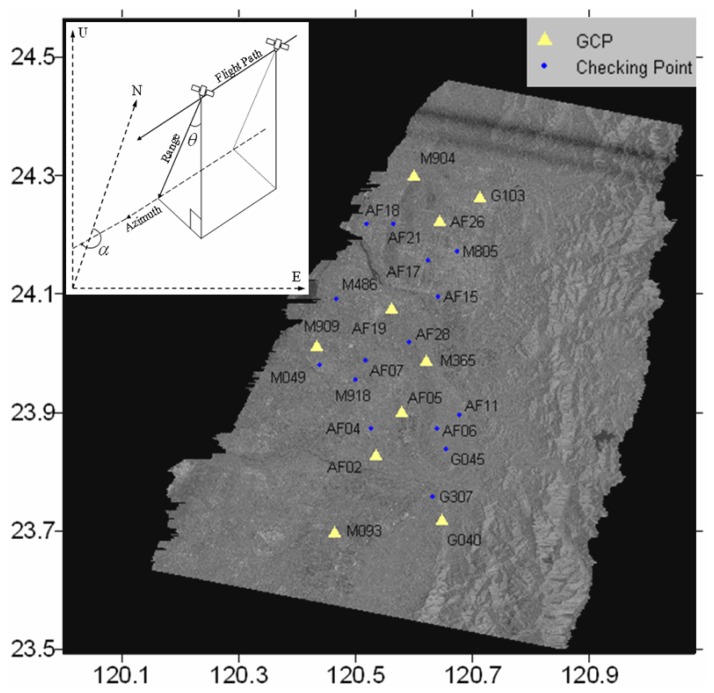
GPS stations used in the study. The yellow triangles represent the stations used as GCPs. The blue dots represent the stations used for validating the results. The inset diagram shows the geometry of radar image acquisition.

Ten of the 25 GPS stations in the study area given by Yu *et al.* [7] will be used as the Ground Control Points (GCP) in this study to help to estimate the non-coseismic components (See Figure 4). The 10 GPS stations selected are evenly distributed over the footwall area of the Chelungpu fault to cover well the displacement trend in the area. GPS observations can give three orthogonal components of the co-seismic displacements in the up, northern and eastern directions. Suppose that the vector *r* = [*r_u_ r_n_ r_e_*]*^T^* represents the three-dimensional (3D) displacements at a GPS site, the displacements vector can be converted into azimuth and range displacements *R_defo_* = [*R_AZO_ R_RO_*]*^T^* following [20]

(3) $$R_{\text{defo}}=U\cdot r$$

where *U* is a matrix that consists of two unit projection vectors, one for the azimuth direction and the other for the range direction

(4) $$U=\left[\begin{array}{ccc} 0 & \cos\alpha & \sin\alpha \\ \cos\theta & \sin\alpha \cos\theta & -\cos\alpha \sin\theta \end{array}\right]$$

where *θ* is the radar incidence angle at the scattering target; and *α* is the azimuth angle of the satellite flight path (clockwise from the North). In the calculations, we define the up, northern and eastern directions as positive for *r_u_*, *r_n_* and *r_e_*. Similarly, we define the satellite flight direction as positive for *R_AZO_*, and the direction towards the satellite from the ground as positive for *R_RO_* .

**Figure 5.**
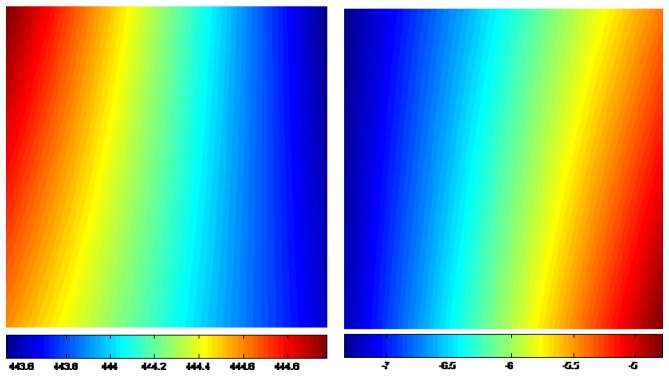
The non-coseismic components of AZO (left) and RO (right) estimated from the GPS observations. Units: m.

The non-coseismic component *R_orbit_* in Equation (1) at the 10 GCPs can be calculated with the azimuth and range displacements of the GCPs. The *R_orbit_* values thus calculated are then used to determine the coefficients *a*_0_, *a*_1_ , *a*_2_ and *a*_3_ in Equation (2) in a least-squares solution. The coefficients are estimated for AZO and RO separately. The non-coseismic components thus obtained for the whole images are shown in Figure 5. Topographic variations can also cause non-coseismic range offsets. However, as such offsets are difficult to model and the baseline of the SAR pair is only 223 m, the effect is neglected in this study.

## 3. Results

**Figure 6.**
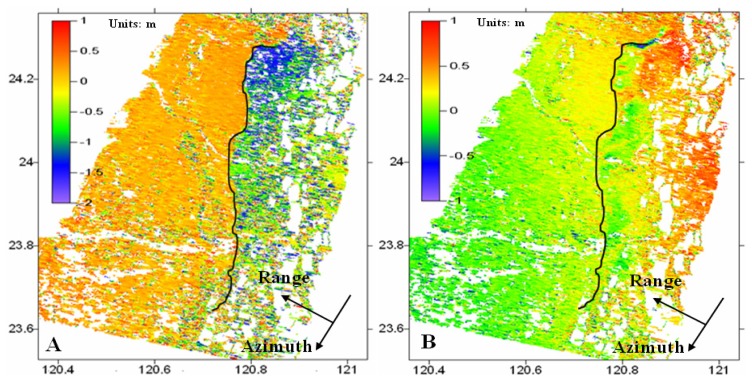
The ground surface displacements in (a) the azimuth and (b) the range directions. The black solid line shows the location of the Chelungpu fault.

Once the azimuth and range offsets and their corresponding non-coseismic components are determined, the azimuth and range displacements caused by the Chi-Chi earthquake can be easily computed by using Equation (1). The results are shown in Figure 6.

**Figure 7.**
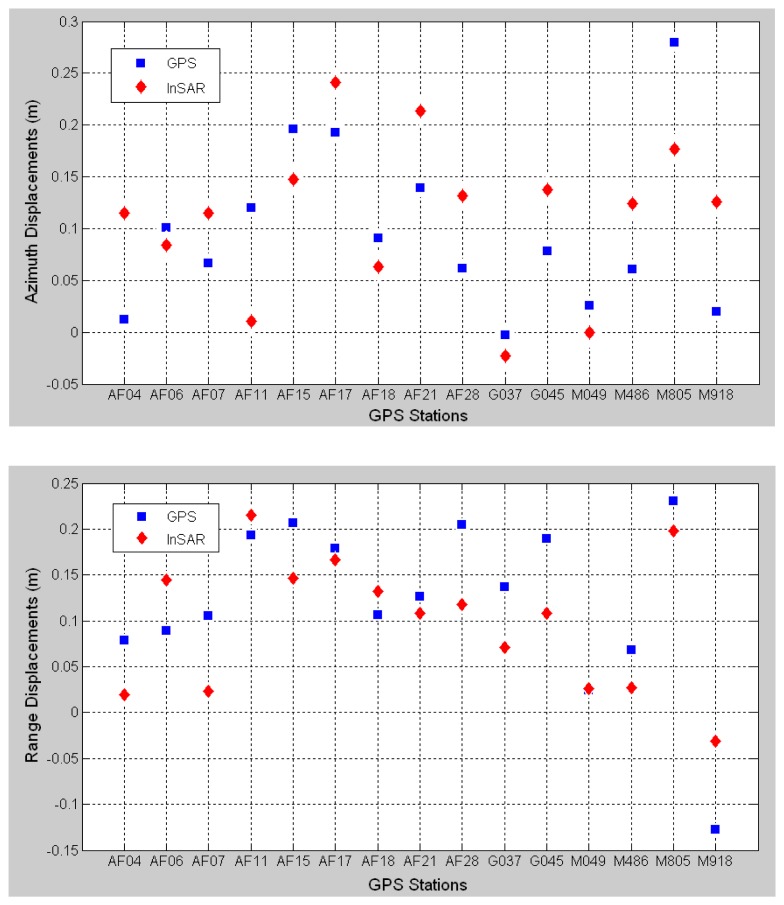
Comparisons between the displacements observed with GPS and from the method of amplitude image matching in azimuth (up) and range directions (low). The GPS stations used for the comparison are listed in Figure 4. The RMS values of the differences between the results are 6.9 cm and 5.7 cm, respectively, in the two directions.

Figure 6A shows the ground displacements in the azimuth direction. It is clear that the co-seismic displacements range from about -2.0 m to 0.7 m in this direction, with the footwall of the fault moving southwards and the hanging wall northwards. The results are very close to those derived from GPS measurements, with a mean discrepancy of less than 10 cm (see Figure 7). However, the displacement in the azimuth direction is difficult to highlight by the DInSAR method as it is almost perpendicular to the LOS direction. The recently developed sub-aperture InSAR method can potential help to determine the displacements in azimuth direction [23-24], however it will not be discussed here as it beyonds the scope of this research. The location of the Chelungpu fault can be easily identified in the displacement maps as the displacements on the two sides of the fault are largely in the opposite directions. The azimuth displacements in the hanging all are well over 0.5m, indicating that large-scale displacements have happened to the hanging wall of the fault.

The ground displacements in the range direction shown in Figure 6B indicates that the earthquake caused displacements varying from about -0.5 m to 1.0 m in this direction. However, only the displacements in the footwall of the fault, i.e., about 30 cm, have been measured in this direction by the DInSAR method (see Figure 2, and [3-5]). The displacements in the hanging wall of the fault, i.e., ∼50 cm, have never been measured with the DInSAR method as the severe decorrelation effect rendered the DInSAR method unusable in the area. Therefore, it is clear that the amplitude image matching method in this case offers much better information on the co-seismic ground displacements although in general the accuracy of the method is not as high as the DInSAR method due to the very low resolution of the ERS SAR images [18].

Fifteen additional GPS stations in the area are used to validate the results (See Figure 4). The up, northern and eastern components of the co-seismic displacements measured at the GPS stations are converted into the azimuth and range displacements and compared with those extracted from Figure 6. It can be seen from Figure 7 that the results from the two methods agree well with each other with the RMS values of the differences between the results being 6.9 cm and 5.7 cm in the azimuth and the range directions respectively.

## 4. Conclusions

A complete two-dimensional co-seismic ground deformation filed associated with the 1999 Chi-Chi earthquake in Taiwan has been generated from two descending ERS-2 SAR images with the SAR amplitude image matching method. DInSAR approach has not worked well in the area due to the severe decorrelation effect in the heavily vegetated mountainous regions. In addition, the DInSAR method has not been able to provide information on the displacements that perpendicular to the radar LOS direction, although the direction is important in this study as the Chelungpu fault trace is in the direction. The SAR amplitude image matching method on the other hand has worked very well in the study area. With the assistance of 10 GPS stations in the area, the co-seismic surface displacements in both the azimuth and range directions have been extracted successfully. Comparisons with the displacements observed at other 15 GPS stations have shown that the RMS values of the differences between the two types of results are 6.9 cm and 5.7 cm in the azimuth and range directions respectively. It is unfortunate that there are no suitable ascending ERS SAR images. Otherwise a 3D surface displacement field may be determined with the method and the least squares approaches [21].

## Figures and Tables

**Table 1.** ERS-2 SAR data used.

**No.**	**Date**	**Orbit**	**Frame**	**Track**

1	15 July 1999	22130	3123	232
2	28 October 1999	23633	3123	232
